# Loss of protein kinase D activity demonstrates redundancy in cardiac glucose metabolism and preserves cardiac function in obesity

**DOI:** 10.1016/j.molmet.2020.101105

**Published:** 2020-10-21

**Authors:** Kirstie A. De Jong, Liam G. Hall, Mark C. Renton, Timothy Connor, Sheree D. Martin, Greg M. Kowalski, Christopher S. Shaw, Clinton R. Bruce, Kirsten F. Howlett, Sean L. McGee

**Affiliations:** 1 Institute for Mental and Physical Health and Clinical Translation, Metabolic Research Unit, School of Medicine, Deakin University, Geelong, Australia; 2 Institute for Physical Activity and Nutrition, School of Exercise and Nutrition Science, Deakin University, Geelong, Australia; 3 Institute of Experimental Cardiovascular Research, University Medical Center Hamburg-Eppendorf, Germany

**Keywords:** Protein kinase D, Cardiac glucose metabolism, Stable isotope metabolomics, Cardiac function, Obesity, Metabolic redundancy

## Abstract

**Objective:**

Protein kinase D (PKD) signaling has been implicated in stress-induced cardiac remodeling and function as well as metabolic processes including contraction-mediated cardiac glucose uptake. PKD has recently emerged as a nutrient-sensing kinase that is activated in high-lipid environments, such as in obesity. However, the role of PKD signaling in cardiac glucose metabolism and cardiac function in both normal and obese conditions remains unknown.

**Methods:**

A cardiac-specific and inducible dominant negative (DN) PKD mouse model was developed. Echocardiography was used to assess cardiac function, while metabolic phenotyping was performed, including stable isotope metabolomics on cardiac tissue in mice fed either regular chow or a high-fat diet (43% calories from fat).

**Results:**

Cardiac PKD activity declined by ∼90% following DN PKD induction in adult mice. The mice had diminished basal cardiac glucose clearance, suggesting impaired contraction-mediated glucose uptake, but normal cardiac function. In obesity studies, systolic function indices were reduced in control mice, but not in cardiac DN PKD mice. Using targeted stable isotope metabolomic analyses, no differences in glucose flux through glycolysis or the TCA cycle were observed between groups.

**Conclusions:**

The data show that PKD contributes to cardiac dysfunction in obesity and highlight the redundancy in cardiac glucose metabolism that maintains cardiac glucose flux *in vivo.* The data suggest that impairments in contraction-mediated glucose uptake are unlikely to drive cardiac dysfunction in both normal and metabolic disease states.

## HIGHLIGHTS

• Cardiac protein kinase D (PKD) is required for contraction-mediated glucose uptake.
• PKD is not essential for normal cardiac function.
• Loss of PKD activity does not alter cardiac glucose flux in normal or obese mice.
• Loss of cardiac PKD activity preserves cardiac function in obesity.

## Abbreviations

[^3^H]-2-DG: 2-[1,2–^3^H]-deoxyglucose
[^3^H]-2-DG6P6: 2-[1,2–^3^H]-deoxyglucose-6-phosphate
ACC: acetyl-CoA carboxylase
αMHC: alpha myosin heavy chain
AUC: area under the curve
Ca^2+^: calcium
CO: cardiac output
DG: diacylglycerol
DN: dominant negative
D_t_: deceleration time
ER: estrogen receptor
GC–MS: gas chromatography mass spectrometry
GLUT4: glucose transporter isoform 4
HFD: high-fat diet
IVSd: end-diastolic intraventricular septum thickness
IVSs: end-systolic intraventricular septum thickness
LV: left ventricle
LVIDd: end-diastolic LV internal diameter
LVIDs: end-systolic LV internal diameter
LVPWd: end-diastolic LV posterior wall thickness
LVPWs: end-systolic LV posterior wall thickness
MPE: molar percent excess
OGTT: Oral glucose tolerance test
PDH: pyruvate dehydrogenase
PDK: pyruvate dehydrogenase kinase
PKD: protein kinase D
ROS: reactive oxygen species
SV: stroke volume

## 1 Introduction

Metabolic diseases such as obesity and type 2 diabetes have profound effects on cardiac metabolism and function that can lead to heart failure [1]. Patients with metabolic diseases are characterized by initial impairments in diastolic function, followed by left ventricular hypertrophy and often impaired systolic function. This form of cardiomyopathy occurs independently of hypertension and is thought to be precipitated by altered cardiac metabolism [2,3]. Although the majority of ATP generation in the heart is derived from fatty acid oxidation, in metabolic disease states, the contribution of glucose oxidation is thought to be further reduced, which impacts cardiac function [4]. While insulin resistance and defects in pyruvate oxidation by pyruvate dehydrogenase (PDH) have been implicated in this response [[5], [6], [7], [8]], the exact mechanisms involved remain unknown. Identifying molecules involved in metabolic and functional alterations of the heart in obesity and type 2 diabetes is critical to address this clinical condition.

Protein kinase D (PKD) signaling is an important regulatory node in both the physiology and pathophysiology of the heart. PKD generally refers to PKD1, but PKD2 and PKD3 isoforms also differ slightly in their N-terminal regulatory regions, which influences isoform-specific functions [9]. There is also considerable redundancy between these three PKD isoforms [10,11]. PKD-mediated phosphorylation of cardiac troponin I [12] and cardiac myosin-binding protein [13] controls cardiomyocyte calcium (Ca^2+^) sensitivity and contractility. Furthermore, PKD induces cardiac dysfunction in response to pressure overload, secondary to its role in driving pathological cardiac hypertrophy [14]. PKD signaling also coordinates metabolic functions in the heart, such as contraction-mediated glucose uptake. Knock-out of PKD1 prevented the contraction-induced increase in glucose uptake by cardiomyocytes *in vitro* [15], while overexpression of constitutively active PKD increased glucose uptake *in vivo* [16]. However, whether PKD controls glucose flux through major metabolic pathways and whether this influences cardiac function remain unknown.

PKD signaling is a sensor of high nutrient environments, including in obesity, in a number of tissues [[17], [18], [19]]. PKD isoforms are activated through two distinct mechanisms. Canonical activation involves G-protein coupled receptor-phospholipase C signaling that increases membrane diglycerides (DGs), which bind to cysteine-rich regions within the N-terminus [20]. This causes a conformational change that exposes serine phosphorylation sites within the catalytic domain. Their phosphorylation increases PKD activity that relieves autoinhibition of the C-terminal catalytic domain [21]. Mitochondrial-derived reactive oxygen species (ROS) can also activate PKD through a mechanism that involves multiple phosphorylation sites within the regulatory domain by a number of ROS-sensitive kinases [22]. Consistent with the accumulation of DG and ROS in the heart in obesity [23,24], recent studies have reported increased PKD2 S916 phosphorylation in the heart in obese mice in the fed state [17]. However, the role of PKD signaling in the heart in metabolic diseases such as obesity remains to be clarified. Our own studies have shown that systemic PKD inhibition improves cardiac function in obese *db/db* mice [17]. Because of PKD's role in cardiac glucose uptake, it has also been hypothesized that PKD inhibition in metabolic diseases could be cardioprotective by reducing glucotoxicity [22]. In contrast, constitutive PKD activation prevents cardiac insulin resistance and mitigates against obesity-induced cardiac hypertrophy, despite inducing hypertrophy in lean animals [16]. Given these divergent findings on cardiac PKD signaling's role in metabolic disease states, the present study determined PKD signaling's role in cardiac glucose metabolism, morphology, and function in both lean and obese mice.

## 2 Materials and methods

### 2.1 Mouse model

**Figure 1:**
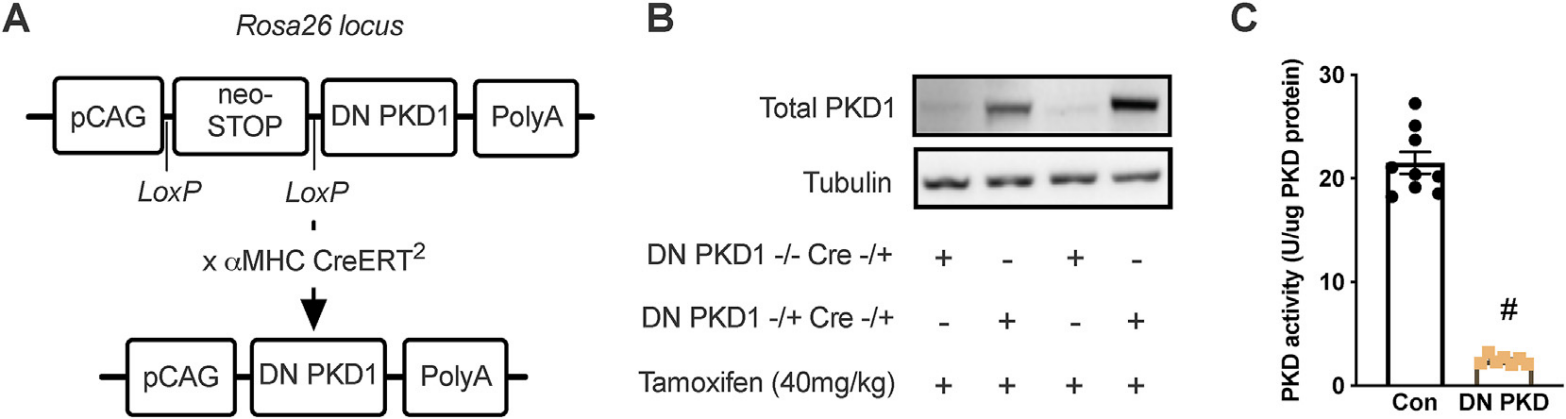
**Induction of DN PKD overexpression reduces PKD activity.** (A) Schematic of the recombined Rosa26 locus with dominant negative (DN) PKD1 knock-in controlled by a pCAG promoter and loxP-flanked STOP codon that was excised with Cre. (B) Total PKD1 protein. (C) PKD activity in the control and cardiac DN PKD mice. Data are mean ± SEM, n = 7–9 mice/group.

A knock-in mouse line expressing a DN PKD1 mutant (K612W) from the Rosa26 locus was generated in collaboration with genOway (Lyon, France). A Rosa26 targeting vector was constructed to express K612W PKD1 with a 3' polyA tail driven by the pCAG promoter with a proximal STOP codon and neomycin resistance gene flanked by loxP sites (Figure 1A). Cre-mediated excision of the STOP codon and neomycin resistance gene overexpressed the DN PKD1 mutant (Figure 1A). The targeting vector was electroporated into C57BL6J ES cells; ∼500 G418-resistant clones were harvested and 30 recombined clones were identified by PCR. These clones were confirmed positive by Southern blotting for both 5' and 3' recombinations. Confirmed recombined ES cell clones were injected into C57BL6J-Tyr^c−2J^/J blastocysts. Chimeric male offspring were mated with C57BL6J females and heterozygous F1 generation knock-in mice were identified by PCR amplification of a region spanning the endogenous Rosa26 locus and the 5' end of the targeting vector. Heterozygous F1 mice were once again mated to C57BL6J mice. The line was subsequently maintained by mating heterozygous DN PKD knock-in mice, to avoid developmental and fertility defects sometimes observed in homozygous Rosa 26-targeted mice [25].

To generate cardiac-specific DN PKD mice, heterozygous DN PKD knock-in mice were mated with mice homozygous for estrogen receptor (ER)-modified Cre driven by α-myosin heavy chain promoter (αMHC-CreERT^2^) obtained from Jackson Laboratories (Sacramento, CA, USA). This modified Cre that is expressed exclusively in cardiomyocytes localized to the nucleus in the presence of tamoxifen to induce recombination [26]. Using this breeding strategy, offspring were all heterozygous for Cre and either null (control) or heterozygous (cardiac DN PKD) for DN PKD knock-in. All of the experiments were conducted in male littermates by directly crossing these two lines. To control for the effects of tamoxifen and activated Cre on cardiac function, all the experimental mice received a single i.p. administration of tamoxifen (40 mg/kg) in 10% ethanol in soybean oil at 14–16 weeks of age. This tamoxifen administration protocol has been found to have no effect on cardiac function in the αMHC-CreERT^2^ line [27]. All the mice were weighed weekly from 12 weeks of age.

### 2.2 Mouse studies

Ethical approval was granted by the Deakin University Animal Ethics Committee (application G32-2014), which is subject to the Australian Code for the Responsible Conduct of Research. One cohort of mice was used to examine DN PKD induction and cardiac PKD activity. Four weeks after tamoxifen administration, the mice were fasted for 5 h before being humanely killed via cervical dislocation. The heart and other tissues were dissected and rapidly frozen. The tissues were analyzed for PKD activity and Western blotting for total PKD as described as follows.

Cardiac function and basal glucose clearance were examined in another cohort of mice. Four weeks after tamoxifen administration, the mice underwent echocardiography under isoflurane anesthesia as described as follows. One week later, the mice underwent glucose clearance studies to determine the role of PKD in contraction-mediated glucose uptake *in vivo*. The mice were administered 2-[1,2–^3^H]-deoxyglucose ([^3^H]-2-DG; 200 μCi/kg; PerkinElmer) in PBS via oral gavage after a 5 h fast. Cold glucose was not simultaneously administered to minimize the influence of glucose effectiveness and insulin action so that contraction-mediated glucose uptake could be best assessed. Prior to and 15, 30, and 60 min after [^3^H]-2-DG administration, 15 μL of blood was obtained from the tail and collected in tubes containing 75 μL of 2.75% zinc sulfate solution. Blood glucose was obtained at the same time using a handheld glucometer. At 60 min after [^3^H]-2-DG administration, the mice were humanely killed via cervical dislocation and the heart was rapidly excised, washed in PBS, and frozen in LN_2_ for later analysis of [^3^H]-2-DG clearance as described as follows. Collected blood was treated with 25 μL of 0.3 N barium hydroxide. Following centrifugation, 50 μL of the supernatant was transferred to a vial containing 1.5 mL of scintillation fluid (PerkinElmer).

For obesity studies, another cohort of mice was fed either regular lab chow (Barastoc Rat and Mouse, Ridley Agriproducts) or a high-fat diet (HFD; 43% digestible energy from fat and 20% from sucrose; SF04-001 Specialty Feeds) two weeks after tamoxifen administration starting at 16–18 weeks of age. At 28–30 weeks of age, an oral glucose tolerance test (OGTT) was performed. The mice were administered a bolus of 50 mg of glucose via oral gavage after a 5 h fast. Blood glucose was measured via handheld glucometer from blood obtained from the tip of the tail prior to glucose administration (designated 0 min) and 15, 30, 45, 60, and 90 min after administration. An additional 30 μL of blood was obtained at 0, 15, 30, and 60 min after administration to determine plasma insulin as previously described [28]. At 29–31 weeks of age, body composition was assessed by EchoMRI, and two days later, the mice underwent echocardiography under isoflurane anesthesia. At 31–33 weeks of age, the mice were administered a bolus of 50 mg of [U–^13^C] glucose (Sigma–Aldrich) via oral gavage. The mice were not handled again until the end of the experiment to minimize the impact of handling stress on the experiment and measurements obtained in the OGTT were used to determine dynamic glucose and insulin responses following a 50 mg oral glucose bolus. Sixty minutes after [U–^13^C] glucose administration, the mice were humanely killed by cervical dislocation and the heart was rapidly excised, washed in PBS, and frozen in LN_2_ for later analysis of ^13^C metabolite labeling, western blotting, and gene expression as described as follows.

### 2.3 Echocardiography

Echocardiography was conducted with the M mode and Doppler ultrasound using a Phillips HD15 diagnostic ultrasound system with a 15 mHz linear-array transducer (Phillips Healthcare) as previously described, including calculations of morphological and functional measurements [17]. In addition, stroke volume (SV) was calculated as validated by Tournoux et al. [29]: SV = LVIDd^3^ - LVIDs^3^, where LVIDd and LVIDs are end-diastolic and end-systolic LV internal diameters, respectively. Cardiac output was calculated as the product of SV multiplied by the heart rate.

### 2.4 Glucose clearance

Cardiac glucose clearance was determined as the fractional clearance of [^3^H]-2-DG-6-phosphate ([^3^H]-2-DG6P6) by the heart. Approximately 20 mg of LV tissue was homogenized in 750 μL of d.H_2_O, centrifuged at 3000 *g* for 10 min at 4 °C, and 200 μL of the supernatant was added to 800 μL of d.H_2_O and transferred to a vial containing 4 mL of scintillation fluid. Another 200 μL of the supernatant was loaded into a 5 mL anion exchange column (Pierce) containing 1 mL of AG1-X8 resin (100–200 mesh, acetate form, BioRad), which binds phosphorylated metabolites including [^3^H]-2-DG6P. The columns were washed twice with 2 mL d.H_2_O, which was collected and 1 mL representing unphosphorylated [^3^H]-2-DG was added to a vial containing 4 mL of scintillation fluid. All of the vials including those containing processed plasma underwent scintillation counting (Beckman Coulter, Mount Waverley, Australia). Glucose clearance was calculated using the following equation: [^3^H]-2-DG clearance = ((DPM of supernatant/gm tissue) - (DPM of column elutant/gm tissue))/plasma [^3^H]-2-DG area under the curve.

### 2.5 Stable isotope metabolomics

[U–^13^C] glucose targeted metabolomic analysis of the heart was conducted using gas chromatography mass spectrometry (GC–MS) similar to that previously described [30]. Approximately 25–30 mg of LV tissue was homogenized in 1.2 mL of 3:1 methanol:dH_2_O using a Precellys bead cryomill (−15 °C) before being centrifuged at 15,000 *g* for 5 min at 4 °C. Metabolites were extracted from the supernatant by collecting the aqueous (top) phase followed by adding chloroform and dH_2_O. The aqueous phase was dried in a speed vacuum and derivatized with 25 μL pyridine and 25 μL N-tert-Butyldimethylsilyl-N-methyl-trifluoroacetamide with 1% tert-Butyldimethylchlorosilane (Sigma–Aldrich). The samples were injected (2 μL splitless, purge flow = 50 mL/min, and purge time = 0.5 min) into an Agilent 6890N gas chromatography system connected to a VF 5 ms capillary column with a 10-m inert EZ-guard (30 m, 0.25 mm, and 0.25 μm) and Agilent 5975C mass selective detector (Agilent Technologies, Mulgrave, Australia) with helium as the carrier gas. The electron ionization mode was used to measure all the organic and amino acids in their TBDMS derivatized form via analyzing their pseudo-molecular fragment ions (that is, M−57). Cardiac-free glucose enrichment was analyzed separately via methane positive chemical ionization GC–MS using the MOX-TMS derivatization method [30], which permits analysis of the pseudo-molecular fragment ion of glucose (554–560 m/z and M0-M6). All the metabolites were analyzed in the selected ion monitoring mode. Ion abundances, measured as the area under the chromatogram curve, were determined using a Mass Hunter workstation (Agilent Technologies, Mulgrave, Australia). Using the matrix method and unlabeled mouse heart samples (88), raw isotopomer data were corrected for naturally occurring isotopic background abundance skew. Sum molar percent excess (MPE) data were calculated by adding all the existing labeled isotopomers (for citrate summation of M1-M6).

### 2.6 PKD activity, Western blotting, and gene expression

Molecular analyses were conducted on LV tissue. Global PKD activity was determined with a colorimetric assay using syntide 2 substrate in the absence or presence (10 μM) of CID755673 PKD inhibitor (Santa Cruz Biotechnology) as previously described [31]. Western blotting that included a standard curve with recombinant PKD1 protein was conducted to quantitatively determine the amount of PKD in the samples (Figure S1A). Western blotting was performed using antibodies toward total PDH Eα1, PDH Eα1 phosphorylated at serine 293, PDK kinase 2 (PDK2) and PDK4 (Abcam), total acetyl-CoA carboxylase (ACC), ACC phosphorylated at serine 79 (Cell Signaling Technology), and α-tubulin (Sigma–Aldrich) as previously described [31]. The expression of *Pdk2* and *Pdk4* was determined as previously described, including normalization to the cDNA concentration as determined by Oligreen [28]. The following primers were used for validation over a range of cDNA concentrations: *Pdk2* forward 5'-TTC CCA CCT GTA CCA CAT GC-3' and reverse 5'-GCT GAA GAG CCT CTC GAT CC-3' and *Pdk4* forward 5'-TGT GAT GTG GTA GCA GTA GTC-3' and reverse 5'-ATG TGG TGA AGG TGT GAA GG-3’.

### 2.7 Statistics

Statistical analyses were conducted using GraphPad Prism.

The data were assessed for normality using the Shapiro–Wilk test. Normally distributed data were analyzed using the t-test or two-way ANOVA with Tukey's multiple comparison testing, while non-normally distributed data were analyzed by the Mann–Whitney test and p < 0.05 was considered statistically significant. Time-dependent OGTT data were analyzed by three-way ANOVA. All the data are represented as mean ± SEM.

## 3 Results

### 3.1 Induction of DN PKD overexpression reduces PKD activity

Our novel cardiac DN PKD mouse model was first characterized through molecular analyses. A single administration of tamoxifen induced a robust increase in total PKD1 expression in the hearts of cardiac DN PKD mice harboring one DN PKD allele (Figure 1B), but not in non-cardiac tissues (Figure S1B). This induction of DN PKD was sufficient to reduce PKD activity by ∼90% in the heart lysates of the cardiac DN PKD mice (Figure 1C).

### 3.2 PKD is required for normal basal cardiac glucose clearance but not cardiac function

**Figure 2:**
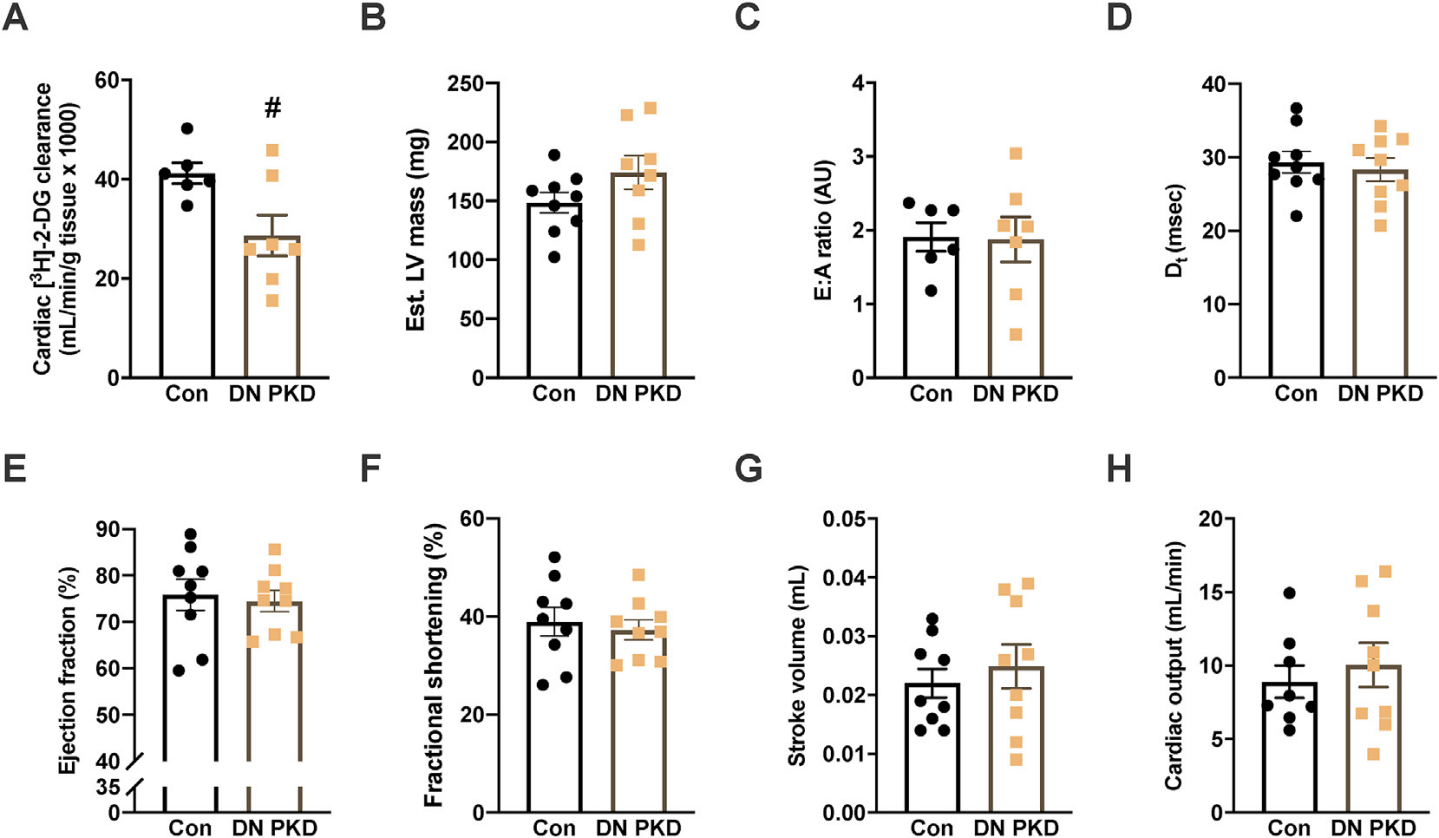
**PKD is required for normal basal cardiac glucose clearance but not cardiac function.** (A) Cardiac 2-[1,2–^3^H]-deoxyglucose ([^3^H]-2-DG) clearance. (B) Estimated left ventricle (LV) mass. (C) E:A ratio. (D) Deceleration time (D_t_). (E) Ejection fraction. (F) Fractional shortening. (G) Stroke volume. (H) Cardiac output in the control and cardiac DN PKD mice. Data are mean ± SEM, n = 7–9 mice/group. Data are mean ± SEM, n = 7–9 mice/group. # denotes p < 0.05 vs control.

PKD's role in cardiac glucose clearance and cardiac function was next assessed. Although blood glucose was lower in the cardiac DN PKD mice throughout the test (Figure S2A), there was no difference in plasma [^3^H]-2-DG throughout the test (Figure S2B). Basal cardiac [^3^H]-2-DG clearance was significantly reduced in the cardiac DN PKD mice (Figure 2A), consistent with previous observations that PKD signaling is involved in contraction-mediated glucose uptake [15,16]. However, there were no differences between the groups in estimated LV mass (Figure 2B) or other morphological indices (Table 1). Furthermore, there were no differences between the groups in measurements of diastolic function, such as the E:A ratio (Figure 2C) and deceleration time (D_t_; Figure 2D). There were also no differences between groups in measurements of systolic function, including ejection fraction (Figure 2E), fractional shortening (Figure 2F), stroke volume (Figure 2G), and cardiac output (Figure 2H). These data suggest that although PKD signaling is required for normal cardiac glucose uptake, it is not essential for normal cardiac function.

**Table 1:** Echocardiography measurements in the control and DN PKD chow-fed mice.

Parameter	Control	DN PKD	P value
Heart rate (beats/min)	432.5 ± 9.6	411.2 ± 13.6	0.306
IVSd (cm)	0.122 ± 0.006	0.136 ± 0.010	0.317
LVIDd (cm)	0.305 ± 0.013	0.316 ± 0.017	0.680
LVPWd (cm)	0.127 ± 0.007	0.141 ± 0.008	0.248
IVSs (cm)	0.172 ± 0.006	0.185 ± 0.011	0.373
LVIDs (cm)	0.189 ± 0.015	0.201 ± 0.016	0.649
LVPWs (cm)	0.138 ± 0.005	0.159 ± 0.009	0.115

Data are mean ± SEM, n = 10–12 mice/group. IVSd, end-diastolic intraventricular septum thickness; LVIDd, left ventricular end-diastolic internal diameter; LVPWd left ventricular end-diastolic posterior wall thickness; IVSs, end-systolic intraventricular septum thickness; LVIDs, left ventricular end-systolic internal diameter; LVPWs, left ventricular end-systolic posterior wall thickness.

### 3.3 Cardiac DN PKD expression does not alter whole body metabolic responses

**Figure 3:**
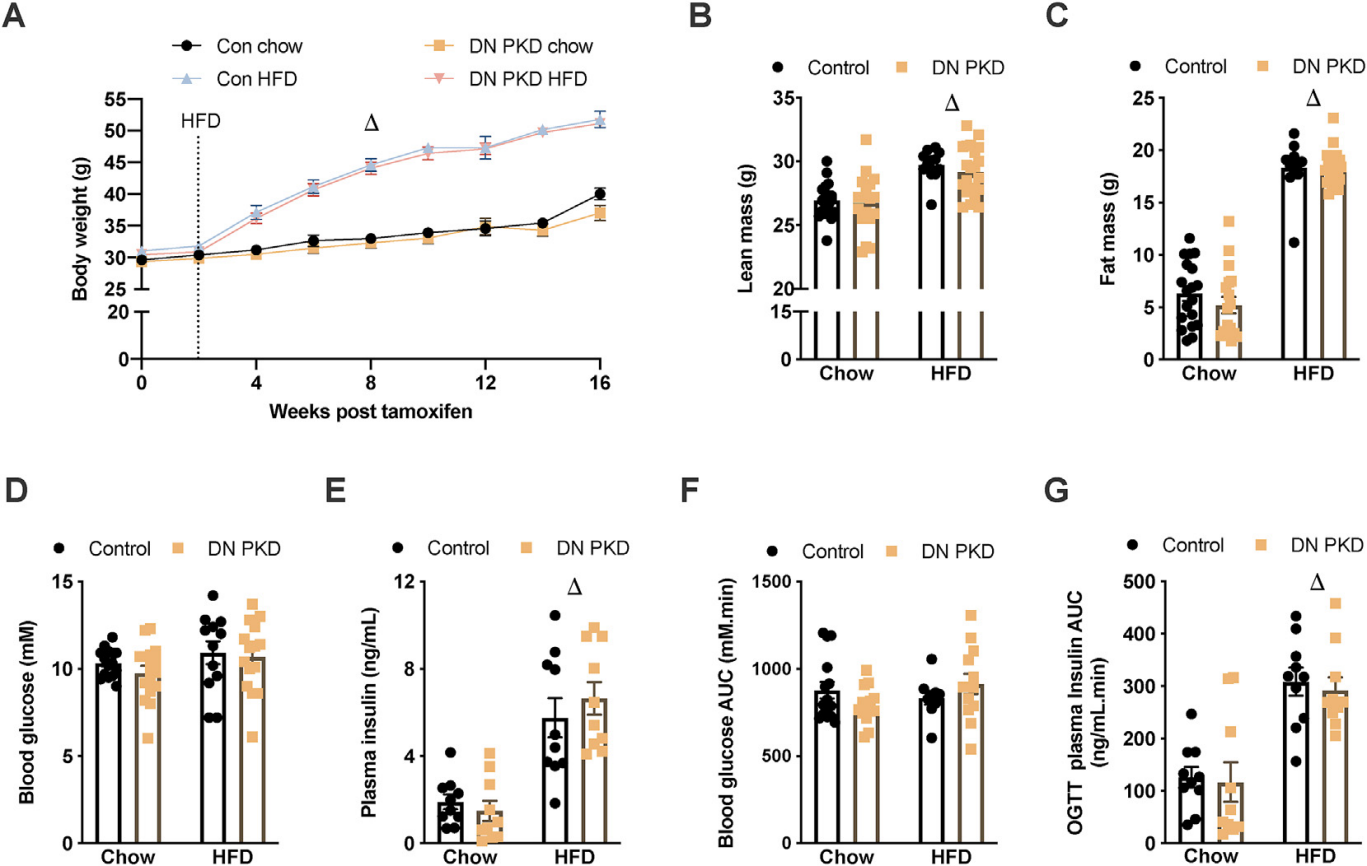
**Cardiac DN PKD expression does not alter whole body metabolic responses.** (A) Body mass. (B) Lean mass. (C) Fat mass. (D) Fasting blood glucose. (E) Fasting plasma insulin. (F) Blood glucose area under the curve (AUC) throughout an oral glucose tolerance test (OGTT). (G) Plasma insulin AUC throughout an OGTT in the control and cardiac DN PKD mice fed either chow or a high-fat diet (HFD). Data are mean ± SEM, n = 12–15 mice/group. Δ denotes the main effect (p < 0.05) of diet.

Before examining cardiac metabolism and function in lean and obese mice, the metabolic phenotype of the control and cardiac DN PKD mice was assessed. The mice fed HFD had significantly higher body mass (Figure 3A), lean mass (Figure 3B), and fat mass (Figure 3C) than the control mice. There was no genotype effect on any of these parameters. There were no differences in fasting blood glucose between the groups (Figure 3D); however, the HFD mice had increased fasting plasma insulin (Figure 3E). There were significant main effects for time and diet and a significant interaction between diet and genotype when analyzing dynamic blood glucose changes throughout the OGTT (Figure S3A). However, there were no differences between groups in blood glucose AUC during the first 60 min of the OGTT (Figure 3F), the time point that was used to examine cardiac glucose flux. There were significant main effects for time and diet when analyzing dynamic plasma insulin changes throughout the OGTT (Figure S3B) and a main effect for diet in plasma insulin AUC during the OGTT (Figure 3G). These data showed that plasma insulin increased in the obese mice, but there were no effects of cardiac DN PKD expression on whole body metabolic responses in the lean and obese mice.

### 3.4 Loss of PKD activity preserves cardiac function in obesity

**Figure 4:**
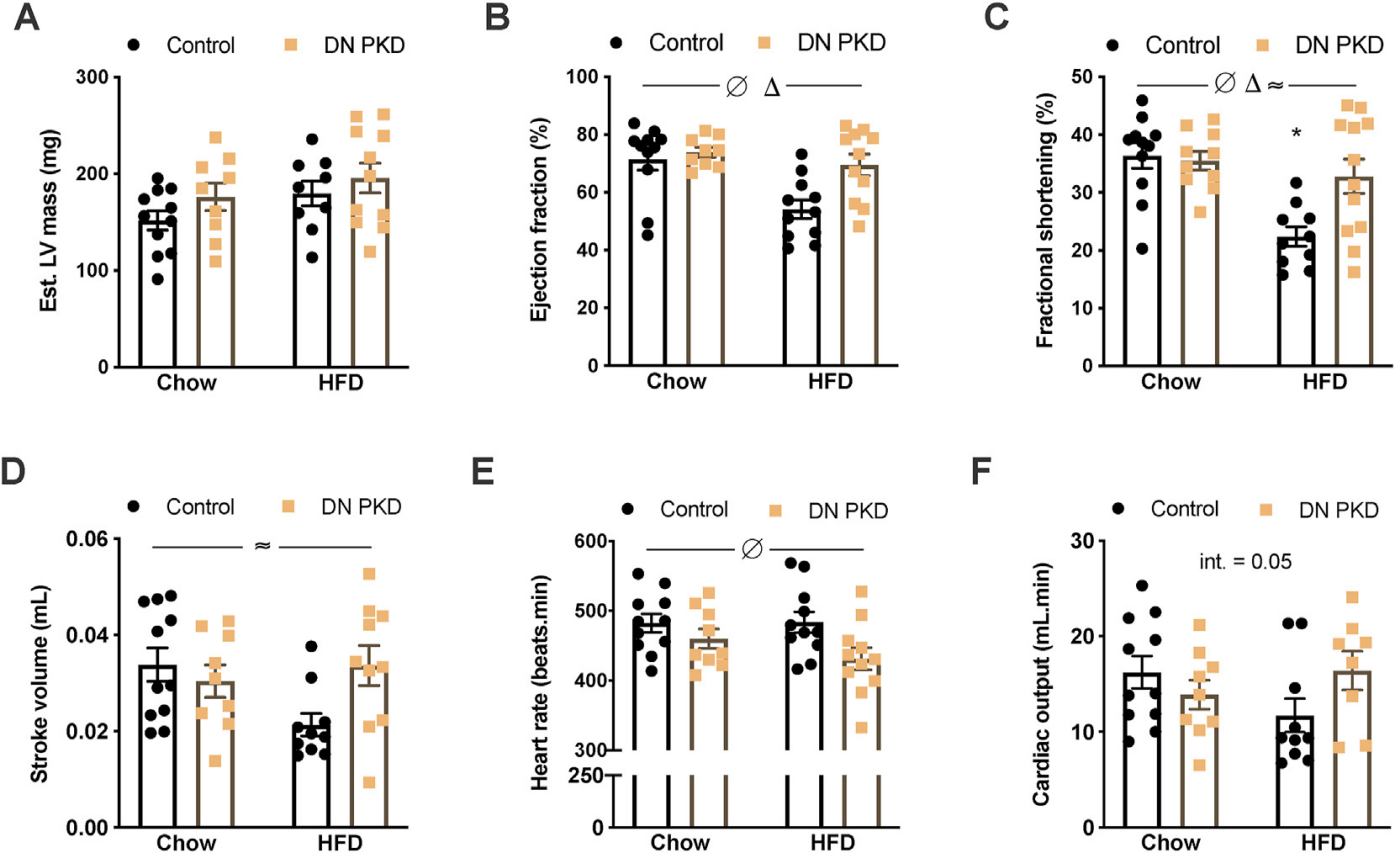
**Loss of PKD activity preserves cardiac function in obesity.** (A) Estimated left ventricle (LV) mass. (B) Ejection fraction. (C) Fractional shortening. (D) Stroke volume. (E) Heart rate. (F) Cardiac output in the control and cardiac DN PKD mice fed either chow or a high-fat diet (HFD). Data are mean ± SEM, n = 9–12 mice/group. Δ denotes the main effect (p < 0.05) of diet. ∅ denotes the main effect (p < 0.05) of genotype. ≈ denotes significant diet x genotype interaction (p < 0.05). ∗ denotes p < 0.05 vs all the other groups.

The role of PKD in cardiac function in obesity was next assessed. PKD activity remained reduced in the obese cardiac DN PKD mice (Figure S4). Although we were unable to obtain reliable mitral Doppler images to assess diastolic function in many of these highly obese mice, M-mode echocardiography did not reveal any differences in estimated LV mass (Figure 4A). However, end-systolic LV posterior wall thickness increased in the DN PKD mice, while end-systolic LV internal diameter increased in the HFD mice (Table 2). Significant diet and genotype interactions were also found for end-diastolic LV posterior wall thickness (LVPWd) and end-systolic intraventricular septum thickness (IVSs; Table 2). Specifically, the cardiac DN PKD chow mice had increased LVPWd compared with the control chow mice (Table 2), which could indicate early LV hypertrophy. In contrast, IVSs was reduced in the cardiac DN PKD chow mice compared with the control chow mice (Table 2). In the control mice, IVSs was reduced by HFD, while in the cardiac DN PKD mice, IVSs was increased by HFD (Table 2), suggesting improved systolic function in the DN PKD mice in obesity. This was directly assessed by quantifying ejection fraction, which was reduced in the obese mice but increased in the DN PKD mice (Figure 4B), and fractional shortening, which was specifically reduced in the control HFD mice compared with all of the other groups (Figure 4C). These data suggest that loss of PKD activity preserves cardiac function in obesity. This was further supported by a significant diet and genotype interaction in the stroke volume assessment (Figure 4D). Heart rate was lower in the cardiac DN PKD mice (Figure 4E) and there was a trend of a diet and genotype interaction for cardiac output (Figure 4F). Collectively, these data suggest that PKD activity is required for cardiac function impairment in obesity.

**Table 2:** Echocardiography measures in the control and DN PKD HFD-fed mice.

Parameter	Chow		HFD		2-way ANOVA p value		
	Con	DN PKD	Con	DN PKD	Gen.	Diet	Int.
IVSd (cm)	0.124 ± 0.006	0.116 ± 0.007	0.128 ± 0.007	0.134 ± 0.005	0.7436	0.0894	0.2695
LVIDd (cm)	0.367 ± 0.016	0.329 ± 0.016	0.354 ± 0.016	0.394 ± 0.026	0.9796	0.1922	0.0621
LVPWd (cm)	0.104 ± 0.005	0.141 ± 0.008∗	0.143 ± 0.010	0.122 ± 0.006	0.3758	0.2294	0.0006
IVSs (cm)	0.201 ± 0.009	0.169 ± 0.008∗	0.164 ± 0.006^+^	0.200 ± 0.008^+^	0.6921	0.8376	0.0002
LVIDs (cm)	0.231 ± 0.016	0.210 ± 0.010	0.274 ± 0.017	0.272 ± 0.027	0.5417	0.0116	0.6719
LVPWs (cm)	0.160 ± 0.008	0.183 ± 0.006	0.157 ± 0.010	0.171 ± 0.005	0.0340	0.3997	0.5820

Data are mean ± SEM, n = 12 mice/group. IVSd, end-diastolic intraventricular septum thickness; LVIDd, left ventricular end-diastolic internal diameter; LVPWd, left ventricular end-diastolic posterior wall thickness; IVSs, end-systolic intraventricular septum thickness; LVIDs, left ventricular end-systolic internal diameter; LVPWs, left ventricular end-systolic posterior wall thickness. Gen., genotype; Int., interaction. ∗p < 0.5 vs control group from the same diet; ^+^p < 0.05 vs the same genotype from the different diet.

### 3.5 Loss of PKD activity does not alter glucose flux through major metabolic pathways

**Figure 5:**
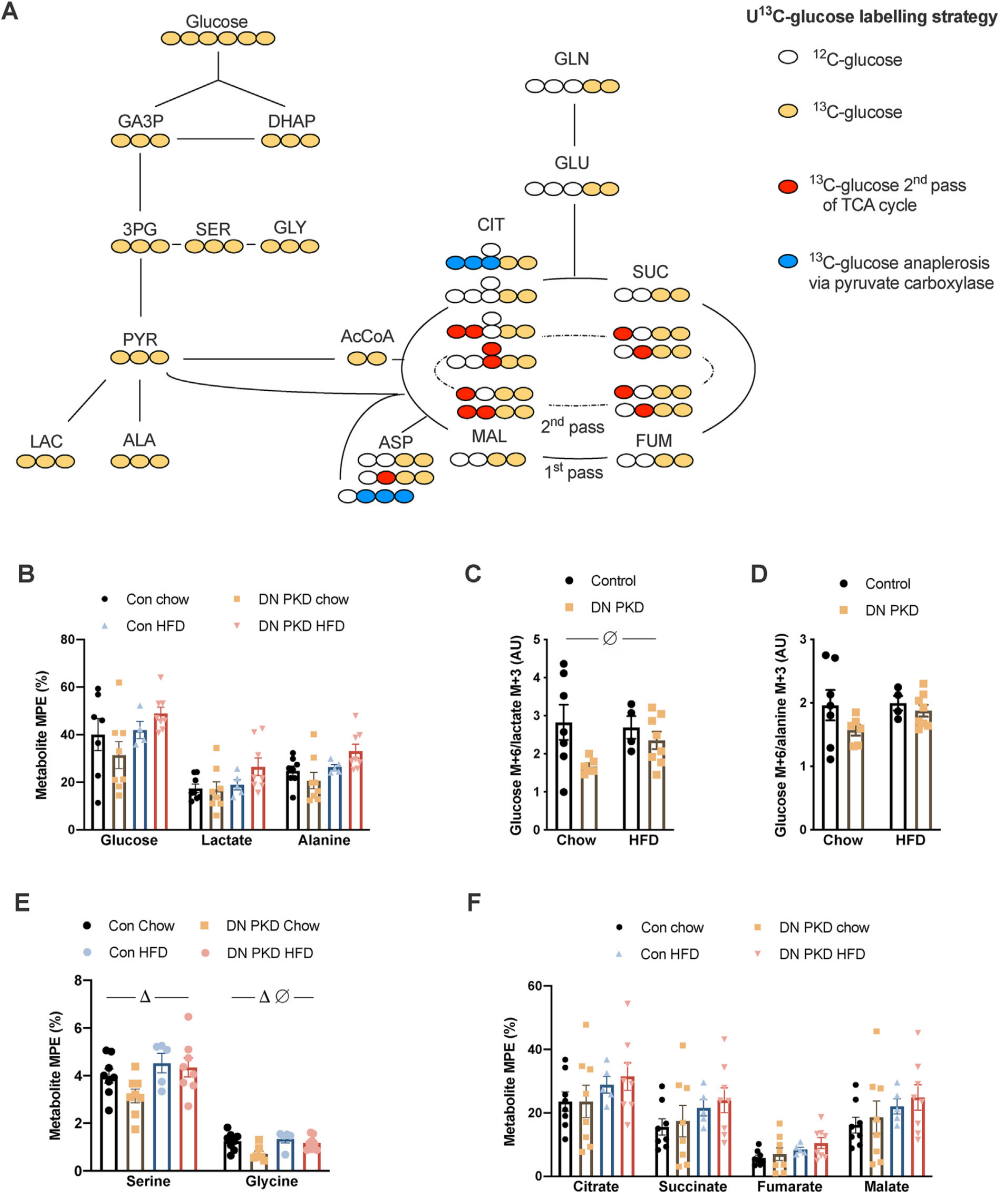
**Loss of PKD activity does not alter glucose flux through major metabolic pathways.** (A) The [U–^13^C] glucose labeling strategy employed to probe cardiac glucose flux. (B) molar percent excess (MPE) of ^13^C labeling of free glucose, lactate, and alanine. (C) Glucose M+6/lactate M+3 ratio. (D) Glucose M+6/alanine M+3 ratio. (E) MPE ^13^C labeling of serine and glycine. (F) MPE ^13^C labeling of citrate, succinate, fumarate, and malate in the control and cardiac DN PKD mice fed either chow or a high-fat diet (HFD). Data are mean ± SEM, n = 5–8 mice/group. Δ denotes the main effect (p < 0.05) of diet. ∅ denotes the main effect (p < 0.05) of genotype.

Manipulation of PKD-regulated glucose metabolism has been hypothesized to be a potential therapeutic approach to combat cardiac dysfunction in obesity [22]. To determine whether the reduction in basal glucose clearance observed in the cardiac DN PKD mice impacted glucose flux in response to a glucose bolus, stable isotope metabolomics analyses were conducted using a U^13^C-glucose labeling strategy (Figure 5A). There were no differences in labeling of cardiac-free glucose, lactate, or alanine between the groups (Figure 5B). As alanine exchanges with pyruvate, both lactate and alanine are representative of flux through glycolysis, so the coupling of free glucose labeling with the labeling of these metabolites was further examined. Notwithstanding the potential contribution of extracellular metabolite exchange and pathway cycling to metabolite labeling around the pyruvate node in the heart [32], there was greater coupling of glucose to lactate labeling, represented by a reduced glucose M+6/lactate +3 ratio, in DN PKD mice (Figure 5C), while there was no difference in the glucose M+6/alanine M+3 ratio between groups (Figure 5D). This suggests a subtle reprogramming of glucose metabolism in cardiac DN PKD mice to maintain glycolytic flux and energetic and/or redox balance. Further supporting this concept, glycine labeling was lower in the cardiac DN PKD mice, while serine and glycine labeling was higher in the HFD mice (Figure 5E). Alanine freely exchanges with pyruvate and is highly correlated with mitochondrial pyruvate [33]. Consistent with similar alanine labeling between the groups, there were also no differences in labeling of the TCA cycle intermediates citrate, succinate, fumarate, and malate (Figure 5F). Consistent with previous findings using this approach *in vivo* [30], there was no effect of diet on the labeling of TCA cycle metabolites. These findings suggest that glucose flux through major ATP producing pathways is not altered in the absence of PKD activity.

### 3.6 Loss of PKD activity does not alter pyruvate anaplerosis

**Figure 6:**
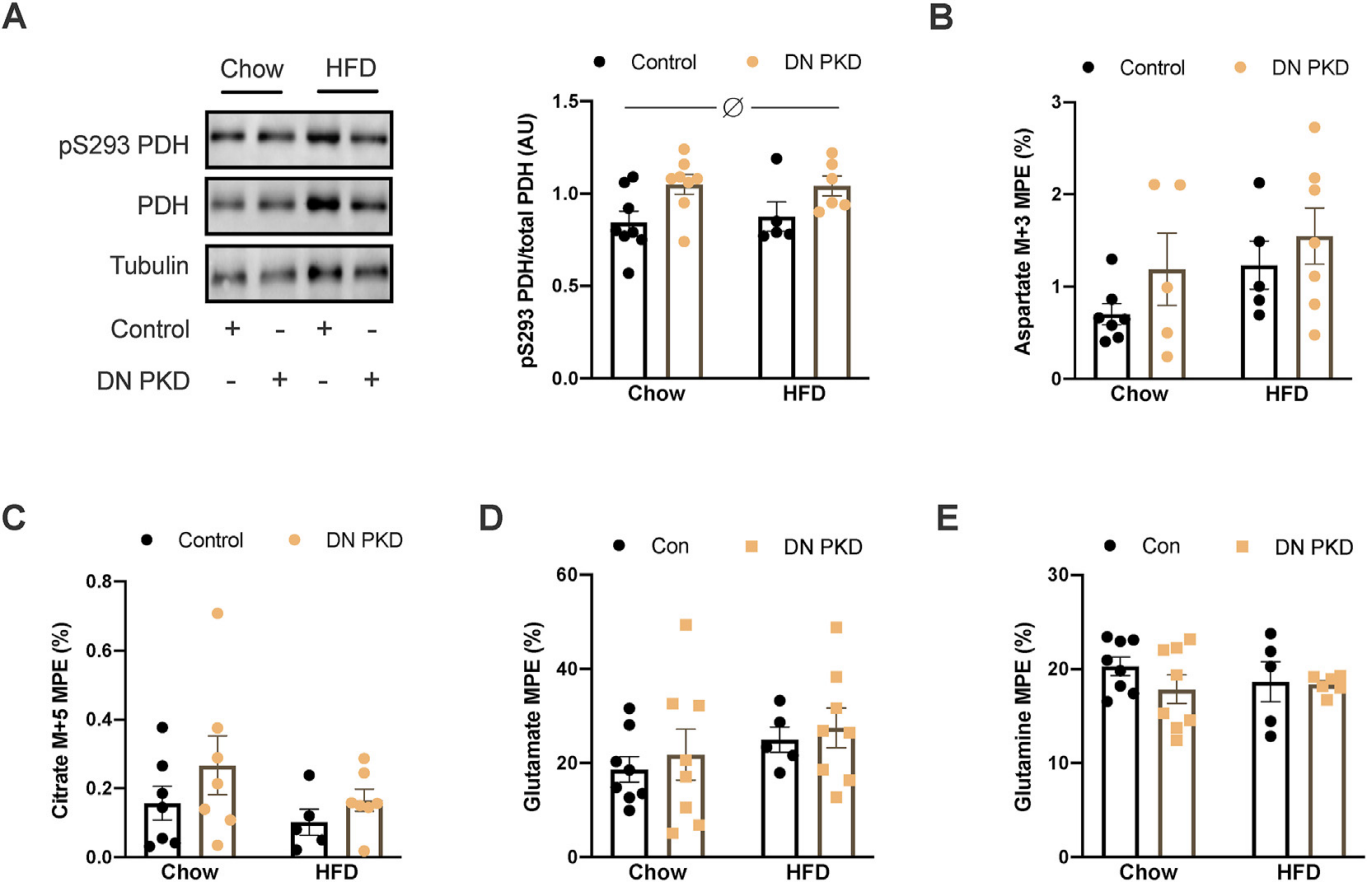
**Loss of PKD activity does not alter pyruvate anaplerosis.** (A) Pyruvate dehydrogenase (PDH) subunit Eα1 phosphorylation at serine 293 relative to the total PDH Eα1 levels. (B) MPE ^13^C labeling of aspartate M+3. (C) MPE ^13^C labeling of citrate M+5. (D) MPE ^13^C labeling of glutamate. (E) MPE ^13^C labeling of glutamine in the control and cardiac DN PKD mice fed either chow or a high-fat diet (HFD). Data are mean ± SEM, n = 5–8 mice/group. ∅ denotes the main effect (p < 0.05) of genotype.

In addition to its involvement in cardiac glucose uptake, PKD has also been implicated in regulating cardiac fatty acid metabolism [34]. Therefore, signaling mechanisms that control the oxidation of fatty acids and glucose were examined. There were no differences in the phosphorylation of acetyl-CoA carboxylase (ACC; Figure S5A), which regulates mitochondrial fatty acid import via CPT-1 [35]. However, PDH phosphorylation at serine 293 of the E1α subunit, which reduces PDH activity [7], was significantly higher in the cardiac DN PKD mice (Figure 6A). There were no genotype differences in the mRNA and protein levels of PDK2 and PDK4 (Figure S5B-E), two of the major PDH kinases in cardiomyocytes [36], which might explain the increased PDH phosphorylation in the cardiac DN PKD mice. However, PDK4 mRNA was reduced and PDK4 protein was increased in the HFD mice (Figure S5D and E). As there were no differences in the labeling of TCA cycle intermediates despite the increase in PDH phosphorylation in the cardiac DN PKD mice, this raised the possibility that pyruvate anaplerosis through pyruvate carboxylase (PC) could increase in the absence of PKD activity. As conversion of pyruvate to oxaloacetate by PC equilibrates with aspartate, M+3 labeling of aspartate is an index of pyruvate anaplerosis (Figure 5A) [33]. However, there were no differences in M+3 labeling of aspartate between the groups (Figure 6B). As multiple TCA cycle passes can also result in M+3 labeling of aspartate (Figure 5A), M+5 labeling of citrate was examined, which results when PC-derived oxaloacetate combines with PDH-derived acetyl-CoA (Figure 5A) [33]. There were no differences between the groups in M+5 labeling of citrate (Figure 6C). Furthermore, the percentage labeling of M+5 citrate was low, which suggests minimal contribution of pyruvate anaplerosis to TCA cycle flux under these conditions. There were also no differences in the labeling of glutamate (Figure 6D) and glutamine (Figure 6E). Together, these data suggest that PKD and obesity does not influence TCA cycle anaplerosis and cataplerosis.

## 4 Discussion

Cardiac glucose metabolism plays a key role in regulating cardiac morphology and function, which are negatively impacted by metabolic diseases such as obesity and type 2 diabetes. The present study defined PKD signaling's role in these processes in both normal and obese mice. We showed that PKD signaling is essential for normal basal glucose clearance, consistent with its role in contraction-mediated glucose uptake [15,16], but has no effect on glucose flux through major metabolic pathways following glucose ingestion. This reveals redundancy in cardiac glucose metabolism under these conditions. In the obese mice, cardiac function was preserved in the absence of PKD signaling, without any effect on glucose flux or cardiac morphology, indicating that PKD signaling is deleterious for the heart in the context of obesity.

At rest, the contracting heart generates ∼30–60% of its ATP from glucose [37,38], and glucose transport is rate limiting for glucose metabolism in cardiomyocytes [39]. Therefore, it is thought that glucose transport capacity is essential for normal cardiac function [40]. Indeed, reduced glucose uptake by the heart is thought to be a precipitating event for impairments in cardiac function that occur in obesity and type 2 diabetes [40]. Our data reinforce that PKD signaling is essential for normal glucose clearance under basal conditions when contraction-mediated glucose uptake is an important contributor to total glucose uptake [41]. However, our findings also showed that there is considerable redundancy in cardiac glucose metabolism. Despite a defective contraction-mediated glucose uptake system, there were no differences in labeling of cardiomyocyte-free glucose following glucose ingestion that induced an insulin response. Cardiac glucose uptake is regulated by distinct mechanisms in response to contraction and insulin, which both converge on GLUT4 translocation to increase glucose uptake. Previous studies found that PKD1 is essential for endosomal GLUT4 vesicle budding [42], which precedes GLUT4 translocation and contraction-mediated glucose transport [15]. While insulin mediated-GLUT4 translocation and glucose uptake also requires endosomal GLUT4 vesicle budding [42], our data suggest that redundant mechanisms could compensate to overcome the defect in contraction-mediated glucose uptake in the absence of PKD signaling. Furthermore, there were no differences in glucose flux through glycolysis and the TCA cycle in the cardiac DN PKD mice in response to an oral bolus of glucose. The tracer strategy used in the present study also revealed insights into additional mechanisms that maintain cardiac glucose utilization. In the DN PKD mice, there was greater coupling of free glucose and lactate labeling, suggesting that glucose flux through glycolysis is prioritized when aspects of the cardiac glucose uptake system are compromised. In support of this idea, glycine labeling, which is a divergent pathway from glycolysis, was reduced in the DN PKD mice. Although flux through this pathway was relatively low, it remains to be determined whether this is also true for other alternate pathways of glucose metabolism, such as glycogenesis and the pentose phosphate pathway. Although there were no differences in labeling of TCA cycle intermediates in the cardiac DN PKD mice, phosphorylation of PDH was higher. Phosphorylation of PDH reduces its activity and is a key mechanism balancing glucose and fatty acid flux into the TCA cycle [7]. In the cardiac DN PKD mice, the increase in PDH phosphorylation, but with normal labeling of TCA cycle intermediates, might have indicated pyruvate anaplerosis through the PC reaction. Increased pyruvate anaplerosis is thought to optimize cardiac fatty acid and glucose oxidation when glucose availability is compromised and has also been linked to enhanced cardiac contractile function [43]. There were trends of pyruvate anaplerotic labeling being higher in the cardiac DN PKD mice, although these were not statistically significant. However, the tracer strategy used in the present study is not optimal for assessing pyruvate anaplerosis and is confounded by multiple passes of the TCA cycle. Future studies using either 3,4-^13^C-glucose or 1-^13^C-pyruvate could give greater clarity on this issue and provide further insights into the subtle reprogramming of cardiac glucose metabolism in the absence of PKD signaling. Interestingly and as we previously observed [30], glucose flux was not impaired in the obese mice. It should be noted however that insulin levels in response to glucose ingestion were significantly higher in the obese mice, suggesting impaired glucose utilization in the obese mice, which required hyperinsulinemia to maintain glucose flux. Collectively, these findings highlight the redundant systems controlling glucose flux through major metabolic pathways and suggest that impaired contraction-mediated glucose uptake and cardiac glucose metabolism more broadly are unlikely to be drivers of impaired cardiac function in obesity.

Obesity had deleterious effects on systolic cardiac function, most notably represented by reduced ejection fraction and fractional shortening, which are hallmark features of progression toward heart failure [44]. In contrast, cardiac function was preserved in the obese cardiac DN PKD mice. This finding is consistent with our previous observation that systemic administration of CID755673 PKD inhibitor enhanced cardiac function in obese mice [17]. The present study revealed that the deleterious effects of PKD on the heart in obesity are cardiomyocyte-specific and occur independently of major alterations in cardiac morphology and glucose metabolism. This suggests that there are as yet unidentified processes by which cardiomyocyte PKD signaling impairs cardiac function in obesity. PKD has been shown to regulate lipoprotein lipase secretion from cardiomyocytes, which increases cardiac lipid accumulation [34]. Other studies suggested that PKD is not required for fatty acid uptake by the heart [15]. Further research is required to determine whether altered fatty acid handling or other mechanisms are involved in the preservation of cardiac function in the absence of PKD signaling in obesity.

In the present study, the decision to develop a DN loss-of-function mouse model was driven by a number of studies employing gene deletion of knockdown approaches that have discovered functional redundancy between the three PKD isoforms. These studies suggest that loss of at least two of the three PKD isoforms is required to ascertain the role of PKD signaling in biological processes. For example, knock-out of both PKD1 and 3, but not each isoform individually, was required to uncover PKD's role in B cell antigen receptor signaling [11]. Similarly, knockdown of both PKD1 and 2 by siRNA was required to identify PKD signaling's role in cell cycle progression [45]. Furthermore, knock-down of PKD1 in cardiomyocytes resulted in a compensatory increase in PKD2 activity [10]. In contrast, the expression of DN PKD mutants has been employed to discover roles of PKD signaling in diverse biological factors both *in vitro* and *in vivo* [[46], [47], [48]]. However, the overexpression of an inactive PKD mutant might induce non-specific interactions with substrates of related kinases, such as PKCs and CaMKs, which could confound interpretation of the data generated from DN PKD experiments [49]. To minimize any nonspecific substrate interactions in our DN PKD model, we used mice that were heterozygous for the DN PKD knock-in allele as our loss of function model, which was sufficient to reduce PKD activity by ∼90% but limited the extent of mutant overexpression. These considerations highlight the complex technical considerations to reflect on when studying kinases with multiple isoforms with high sequence homology.

## 5 Conclusions

In conclusion, we found that a loss of PKD signaling reveals redundancy in cardiac glucose metabolism, which suggests that impairments in contraction-mediated glucose uptake are unlikely to drive cardiac dysfunction in metabolic disease states. Our findings also showed that reducing PKD activity preserves cardiac function in obesity independently of changes in cardiac morphology and glucose metabolism.

## Funding

This study was supported by grants from the 10.13039/501100000971Diabetes Australia Research Program, the 10.13039/501100000925National Health and Medical Research Council of Australia (APP1163238), and the Center for Molecular and Medical Research (10.13039/501100001778Deakin University) to SLM. CRB (grant FT160100017) and GMK (grant DE180100859) are supported by 10.13039/501100000923Australian Research Council fellowships.

## Author contributions

KADJ, KFH, and SLM designed and planned the study. KADJ, LGH, MCR, TC, SDM, and GMK conducted the experiments and analyzed the data. CSS, CRB, KFH, and SLM provided reagents and expertise and interpreted the data. SLM provided funding. SLM wrote the paper and all of the authors edited and approved the final version. SLM is the guarantor of this study and, as such, had full access to all of the study data and takes responsibility for the data integrity and the accuracy of the data analysis.
