# Supplementary material for: Loss of protein kinase D activity demonstrates redundancy in cardiac glucose metabolism and preserves cardiac function in obesity
Source: Mol Metab. 2020 Oct 21;42:101105. doi: 10.1016/j.molmet.2020.101105 (PMC7680779; doi:10.1016/j.molmet.2020.101105)
Supplement: Multimedia component 1 [file mmc1.docx]

**Supplementary information for**

**Loss of protein kinase D activity reveals redundancy in cardiac glucose metabolism and preserves cardiac function in obesity**

Running title: Role of PKD in cardiac metabolism and function

Kirstie A. De Jong^1,3,^, Liam G. Hall^1^, Mark C. Renton^2^, Tim Connor^1^, Sheree D. Martin^1^, Greg M. Kowalski^1,2^, Christopher S. Shaw^2^, Clinton R. Bruce^2^, Kirsten F. Howlett^2^, Sean L. McGee^1*^

^1^Institute for Mental and Physical Health and Clinical Translation, Metabolic Research Unit, School of Medicine, Deakin University, Geelong, Australia; ^2^Institute for Physical Activity and Nutrition, School of Exercise and Nutrition Science, Deakin University, Geelong, Australia; ^3^Institute of Experimental Cardiovascular Research, University Medical Centre Hamburg-Eppendorf, Germany.

**SUPPLEMENTARY FIGURE 1**

**Supplementary Figure 1.** (A) Quantification of PKD protein in heart lysates in Control and cardiac dominant negative (DN) PKD mice. (B) Total PKD in the quadriceps skeletal muscle, epididymal adipose tissue (Epi fat) and the kidney in Control and cardiac dominant negative (DN) PKD mice. Std., standard curve.

**SUPPLEMENTARY FIGURE 2**

**Supplementary Figure 2.** (A) Blood glucose, and; (B) Plasma 2-[1,2-^3^H]-deoxyglucose ([^3^H]-2-DG) following [^3^H]-2-DG administration in Control and cardiac dominant negative (DN) PKD mice. Data are mean ± SEM, n = 6-7 mice/group. ∅ denotes main effect for genotype. ∞ denotes main effect for time.

**SUPPLEMENTARY FIGURE 3**

**Supplementary Figure 3.** (A) Blood glucose, and; (B) Plasma insulin throughout an oral glucose tolerance test (OGTT; 50 mg glucose) in Control and cardiac dominant negative (DN) PKD mice fed either chow or high fat diet (HFD). Data are mean ± SEM, n = 12-15 mice/group. Δ denotes main effect for diet (p<0.05). ∞ denotes main effect for time (p<0.05). ƒ denotes significant genotype x diet interaction (p<0.05).

**SUPPLEMENTARY FIGURE 4**

**Supplementary Figure 4.** Cardiac PKD activity in Control and cardiac dominant negative (DN) PKD mice fed a high fat diet. Data are mean ± SEM, n = 5-7 mice/group. # denotes p < 0.05 vs Control.

**SUPPLEMENTARY FIGURE 5**

**Supplementary Figure 5.** (A) Acetyl-CoA carboxylase (ACC) phosphorylation at serine 79 normalised to total ACC; (B) Pyruvate dehydrogenase kinase 2 (*Pdk2*) gene expression; (C) PDK2 protein; (D) *Pdk4* gene expression, and; (E) PDK4 protein in Control and cardiac dominant negative (DN) PKD mice fed a high fat diet. Data are mean ± SEM, n = 5-7 mice/group. Δ denotes main effect (p < 0.05) for diet.
